# An Intervention to Reduce Residential Insecticide Exposure during Pregnancy among an Inner-City Cohort

**DOI:** 10.1289/ehp.9168

**Published:** 2006-07-27

**Authors:** Megan K. Williams, Dana B. Barr, David E. Camann, Linda A. Cruz, Elizabeth J. Carlton, Mejico Borjas, Andria Reyes, Dave Evans, Patrick L. Kinney, Ralph D. Whitehead, Frederica P. Perera, Stephen Matsoanne, Robin M. Whyatt

**Affiliations:** 1 Columbia Center for Children’s Environmental Health, Mailman School of Public Health, Columbia University, New York, New York, USA; 2 National Center for Environmental Health, Centers for Disease Control and Prevention, Atlanta, Georgia, USA; 3 Southwest Research Institute, San Antonio, Texas, USA; 4 Department of Obstetrics and Gynecology, Columbia University, New York, New York, USA

**Keywords:** insecticides, integrated pest management, intervention, prenatal, residential

## Abstract

**Background:**

We previously reported widespread insecticide exposure during pregnancy among inner-city women from New York City. Here we report on a pilot intervention using integrated pest management (IPM) to reduce pest infestations and residential insecticide exposures among pregnant New York City African-American and Latina women (25 intervention and 27 control homes).

**Methods:**

The IPM consisted of professional cleaning, sealing of pest entry points, application of low-toxicity pesticides, and education. Cockroach infestation levels and 2-week integrated indoor air samples were collected at baseline and one month postintervention. The insecticides detected in the indoor air samples were also measured in maternal and umbilical cord blood collected at delivery.

**Results:**

Cockroach infestations decreased significantly (*p* = 0.016) after the intervention among intervention cases but not control households. Among the intervention group, levels of piperonyl butoxide (a pyrethroid synergist) were significantly lower in indoor air samples after the intervention (*p* = 0.016). Insecticides were detected in maternal blood samples collected at delivery from controls but not from the intervention group. The difference was significant for *trans*-permethrin (*p* = 0.008) and of borderline significance (*p* = 0.1) for *cis*-permethrin and 2-isopropoxyphenol (a propoxur metabolite).

**Conclusion:**

To our knowledge, this is the first study to use biologic dosimeters of prenatal pesticide exposure for assessing effectiveness of IPM. These pilot data suggest that IPM is an effective strategy for reducing pest infestation levels and the internal dose of insecticides during pregnancy.

Insecticide use in inner-city communities in the United States is widespread, and resultant indoor exposures can be extensive (Fenske et al. 1990; Gurunathan et al. 1998; Lemus et al. 1998; Whitmore et al. 1994; Whyatt et al. 2003). In a recent study of African-American, Latina, and Caucasian mothers and newborns residing in East Harlem in New York City, 72% of subjects reported indoor insecticide exposure during pregnancy to control cockroaches and other pests. Maternal urine samples collected on delivery showed that 55% of subjects had detectable levels of 3,5,6-trichloro-2-pyridinol (TCPy), a metabolite of the organophosphate chlorpyrifos, and 37% had detectable levels of 3-phenoxy-benzoic acid, a metabolite of pyrethroid insecticides (Berkowitz et al. 2004). In our prior study of pregnant African-American and Latina women residing in northern Manhattan and the South Bronx, 85% of subjects reported that pest control measures were used during pregnancy, and 100% of participants had detectable airborne exposures to organophosphate and carbamate insecticides (Whyatt et al. 2003). In addition, these same insecticides were detected in 45–74% of blood samples collected from mothers and newborns at delivery. Insecticide levels in maternal and newborn blood samples were similar and highly correlated, suggesting that placental transfer of these compounds can occur (Whyatt et al. 2003). Further, the infants with the highest *in utero* exposure to the organophosphate chlorpyrifos had significantly lowered weight and length at birth and significantly poorer mental and motor development at 3 years of age (Rauh et al., in press; Whyatt et al. 2004). Experimental evidence has linked exposure to organophosphate insecticides during gestation or the early postnatal period to adverse neurodevelopmental sequelae in the offspring (reviewed by both Eskenazi et al. 1999; Landrigan et al. 1999).

Although there is sufficient evidence documenting that residential insecticide use is pervasive among African-American and Latino populations residing in low-income urban communities in New York City, there is a paucity of data in the current literature describing methods to reduce residential insecticide exposure in these environments. Attempts have been made to determine the risk factors associated with pest infestation and resulting insecticide use. A survey of pest control measures used by residents of public housing in New York State conducted during 2000–2001 concluded that pest problems and insecticide use were related to housing disrepair and housing density (Surgan et al. 2002). Additional evidence has shown that cockroaches and other household pests thrive in multifamily dwellings where excessive moisture, cracks and crevices, and abundant food sources are present (Brenner et al. 2003). In our prior research, the proportion of women reporting that pests were sighted in the home, as well as the proportion reporting that pest control measures were used during pregnancy, increased significantly with the level of disrepair in the home (including holes and cracks in the walls and ceilings, water damage, leaky pipes, peeling or flaking paint) (Whyatt et al. 2002). Women who reported sighting pests in their home (primarily cockroaches and rodents) were significantly more likely to use pest control measures than women reporting no pest sightings (chisquare test, *p* < 0.001) (Whyatt et al. 2003). Therefore, effective interventions to reduce pest infestation levels and residential insecticide exposure must address aspects of housing quality such as unrepaired cracks and crevices, leaky pipes, and food sources.

Integrated pest management (IPM) is considered an environmentally sustainable pest control strategy. It aims to reduce pest populations by identifying and understanding the biology and behavior of the insects and rodents; selecting and implementing a set of environmentally safe and effective control strategies; and monitoring the effectiveness of the strategies (Ogg et al. 1995). Techniques include building repairs to eliminate pest entry points and breeding sites, cleaning to remove pest food sources, and the use of low-toxicity, nonaerosol insecticides including baits, gels, and boric acid (Ogg et al. 1995). Interventions that have included IPM-like practices have been endorsed by the National Institutes of Health (1997), and several studies have documented the ability of IPM interventions to reduce pest populations, allergen levels, and asthma morbidity (Arbes et al. 2003; Brenner et al. 2003; Morgan et al. 2004; Wood et al. 2001). However, only limited data address the effectiveness of IPM interventions at reducing residential insecticide exposure (Campbell et al. 1999; Kass and Outwater, 2001).

The current study is the first to use bio-markers and air monitoring to document changes in insecticide exposure after an IPM intervention. The IPM strategy used here was adapted from the Columbia Intervention to Reduce Indoor Allergens Study (Kinney et al. 2002). The aim of this study was to assess the feasibility of reducing prenatal exposures to pests and insecticides through an IPM intervention that included professional cleaning, building repairs, sealing pest entry points, professional insecticide placement, and one-on-one education.

## Study Design and Methods

### Subject recruitment

#### Intervention group

Recruitment and enrollment efforts for the intervention study occurred from August 2002 through April 2004. Thirty women were recruited from obstetrics and gynecology (OB/GYN) clinics located in New York Presbyterian and Harlem Hospitals. Eligibility was restricted to women 18–35 years of age who self-identified as either African American or Latina (Dominican or Puerto Rican) and reported using high-toxicity insecticides (use of exterminators, can sprays, and/or pest bombs) during pregnancy. Further, eligible subjects must have resided in northern Manhattan (north of 110th Street) or the South Bronx (south of Fordham Road) for at least 1 year before pregnancy and must not be planning to move from the community before delivery. From the 30 subjects who completed the screening and consent forms, 5 women dropped from the study between enrollment and monitoring (2 women gave birth before the intervention was completed, 1 experienced health problems restricting her participation, and 2 moved out of the community). Samples collected from the subjects before the intervention will be referred to as preintervention, and samples collected after the intervention will be referred to as postintervention. From the remaining 25 women, 25 (100%) completed the prenatal questionnaire, 25 (100%) participated in pre- and postintervention indoor air monitoring, and biologic samples were collected from 21 (84%) subjects. Nineteen (76%) completed pre- and postintervention assessment of pest infestation.

#### Control group

The control group was selected from participants in an ongoing prospective cohort study designed to validate biomarkers of prenatal insecticide exposure. As part of this study, insecticide levels were measured in 2-week integrated indoor air samples collected continuously over the last 2 months of pregnancy. Blood samples were collected from the mother and newborn at delivery. Enrollment for this study occurred in the OB/GYN clinics located in New York Presbyterian Hospital and Harlem Hospital from October 2001 through July 2004. The recruitment strategy and eligibility criteria for the controls were identical to those for the cases. From the total of 110 women fully enrolled in the biomarker validation study, 27 were selected as controls for the intervention study. Control selection aimed to match case subjects on year of enrollment (2002–2004) and self-reported use of high-toxicity insecticides (use of exterminators, can sprays, and/or pest bombs) during pregnancy. Baseline and follow-up integrated air samples were selected to match the pre- and postintervention samples in the cases. Questionnaire data were available for 100% of subjects. Baseline and follow-up indoor air data were available for 24 (88%) subjects; blood samples were collected from 17 (63%) subjects. Fourteen (52%) subjects completed the initial pest infestation levels. Follow-up samples were available for only six (22%) subjects.

The institutional review board of the Columbia Presbyterian Medical Center approved the study, and we obtained written informed consent from all study subjects.

### Intervention

The intervention commenced at the conclusion of the 2-week preintervention monitoring period. This IPM consisted of three main components: an extensive cleaning with minor building repairs, a low-toxicity insecticide application, and behavioral/health education. The kitchen, bathroom, and living room areas of the intervention apartments were professionally cleaned using low-toxicity, citrus-based cleaning products. Pest entry points were sealed with caulking compounds and/or metal screens. A professional insecticide placement company injected low-toxicity insecticides, 2.15% hydramethylnon (MAXFORCE; Maxforce Insect Control Systems, Oakland, CA), or small amounts of boric acid directly into the cracks and holes before sealing and placed glue traps for cockroaches throughout the kitchen, bathroom, and problem areas. Hydramethylnon has low toxicity and low vapor pressure and has been shown previously to be effective for long-term cockroach control (Appel 1992). Using a checklist prepared for the Columbia Intervention to Reduce Indoor Allergens Study, the health educator assessed the frequency and location of pest sightings and tailored the health education accordingly for each participant. After the cleaning, a health educator met with the family to discuss IPM strategies for pest control. Training sessions targeted as many household members as possible and strongly emphasized a team effort. Strategies included removing garbage from the home each day, eating meals only in the kitchen, and cleaning up dishes and food spills as soon as possible. In addition, the program included education and instruction regarding nontoxic pest control methods. Airtight containers for food and trash storage were provided to each household. The intervention cleaning and behavioral training took place over approximately 2–3 days. The control group did not receive an IPM intervention, nor did they receive a placebo intervention. However, all subjects in the biomarker validation study, including those selected as controls for the current study, received written material on the importance of reducing insecticide use in the home and techniques for controlling pests without using higher toxicity insecticides.

### Sample collection

#### Questionnaire data

A 45-minute questionnaire was administered to the intervention and control groups in each woman’s home by a trained bilingual interviewer during the third trimester of pregnancy. The questionnaire included information on demographics, home characteristics including housing disrepair and pest infestation levels, lifetime residential history, history of active and passive smoking, occupational history, maternal education and income level, alcohol and drug use during pregnancy, and history of residential insecticide use. Information about insecticide use included whether or not any pest control measures were used by an exterminator or by others (the woman herself, other household members, or the building superintendent) during pregnancy and, if so, what types of measures were used and at what frequency (Perera et al. 2003; Whyatt et al. 2002, 2003).

#### Indoor air monitoring

Before the intervention, a baseline 2-week integrated indoor air sample was collected from the homes of subjects in the intervention and control groups. Monitoring commenced in each home at the end of the second or beginning of the third trimester of pregnancy using a BGI pump with a 0.5-L/min flow-rate (BGI, Inc., Waltham, MA). The pump was attached to a URG (University Research Group, Chapel Hill, NC) polyurethrane foam (PUF) sampler with a 2.5-μm inlet cut fitted with a 30-mm quartz fiber filter and a foam cartridge backup to capture semivolatile vapors and aerosols. The pumps were attached to a battery and operated continuously over the 2 weeks. The monitoring equipment was placed in the main living area of the apartment, with the pump in a secure box and the sampler (located inside a protective wire cage) placed at least 60 cm from wall surfaces at a height of 135 cm. The sampler height was chosen to represent the average between the woman’s sitting and standing heights, because residential insecticide air concentrations have been shown to vary with height, being greatest near the floor (Aprea et al. 2000; Fenske et al. 1991). Study subjects were instructed on the importance of not disturbing the equipment and told to go about their daily activity as normal. The research staff returned after 2 weeks to collect the equipment, perform a leak check, and record the pump flow-rates. A careful log was kept of elapsed time on the pump meter and of rotometer readings and leak check results at each visit. The monitoring strategies for intervention and control homes were identical. Prior quality control analyses indicated that there would be no danger of insecticide breakthrough with this monitoring protocol (Camann and Whyatt 2001).

Approximately 4 weeks after the intervention, a follow-up 2-week integrated indoor air sample was collected from intervention and control homes. Protocols for the follow-up air monitoring were identical to those for the baseline sample. The monitoring was targeted to occur during the 38th to 40th week of pregnancy; however, because of premature births or postponements, some subject’s homes were monitored immediately after delivery.

#### Cockroach infestation levels

To monitor cockroach infestation levels, six pheromone glue traps (Victor Roach Pheromone Traps; Woodstream, Lititz, PA) were placed in standardized locations throughout the kitchens of each subject during the 2-week baseline and follow-up indoor air monitorings. After 2 weeks, traps were collected and the number of adult and nymph cockroaches caught in each trap was counted.

#### Maternal and cord blood

We used blood collection procedures validated in our prior research studies to ensure that blood samples (maternal and/or umbilical cord) were collected from women in the intervention and control groups at delivery (Whyatt et al. 2003). A sample of infant cord blood was collected by delivery room staff immediately after the cord was cut and the placenta delivered. Infant cord blood was obtained by syringing the blood into heparinized syringes at the point the cord enters the placenta. A sample of maternal blood (30–35 mL) was obtained within 1–2 days postpartum by the research staff or by hospital staff. A member of the research staff transported the blood samples to the biomarker laboratory located at Columbia University, New York City, immediately after collection. Within 12 hr of blood collection, the cord and maternal bloods were transferred to centrifuge tubes and spun for 15 min at 1,500 rpm. Plasma samples were collected and stored at −70°C before shipment to the Centers for Disease Control and Prevention (CDC) for insecticide analysis.

### Pesticide analysis

#### Pesticides in air monitoring filters

Analysis of insecticides in the 2-week integrated indoor air samples was conducted by Southwest Research Institute under the direction of D. Camann. Immediately after each 2-week collection period, air monitoring filters were brought to the laboratory at the Columbia Children’s Center for Environmental Health, inventoried and stored at 20°C. Every 4–6 weeks, air samples were shipped to Southwest Research Institute. The entire PUF plug and filter was placed in a Soxhlet extractor, spiked with terphenyl-d_14_ as a recovery surrogate, extracted with 6% diethyl ether in hexane for 16 hr and concentrated to 1.0 mL in 10% ether in hexanes. Extracts were stored frozen below −4°C. Insecticides are stable in the extract under these conditions. We determined the amounts of the target insecticides in samples using Agilent 6890/5973 gas chromatography/mass spectrometry (Agilent, Wilmington, DE) in selected ion mode. Paired pre- and postintervention air samples were available on 25 cases and 39 controls. The target insecticides that were measured in the indoor air samples were bendiocarb, carbaryl, carbofuran, *cis-* and *trans-*permethrin, malathion, methyl parathion and propoxur. In addition, piperonyl butoxide, a synergist added to natural and synthetic pyrethroid insecticides, was measured as an indicator of pyrethroid insecticides. Chlorpyrifos and diazinon were not assessed because most of the women were enrolled in the study after the federal ban on their residential use and our prior data indicate that the ban was effective at reducing use and exposures to these insecticides among inner-city women in New York City (Carlton et al. 2004; Whyatt et al. 2003, 2004).

#### Pesticides in plasma samples

Analysis of the insecticides or their metabolites in maternal and cord plasma was conducted by the CDC under the direction of D. Barr using isotope dilution gas chromatography–high resolution mass spectrometry (Barr et al. 2002). Approximately 10–15% of all samples assayed were positive or negative control samples. Two concentrations of positive control samples were used: one spiked at the mid-calibration range and one at the low-calibration range. A set of blinded positive control samples was also run, which an independent quality assurance officer evaluated. CDC provided results on insecticide levels in maternal blood samples for 21 cases and 32 controls and from umbilical cord blood for 13 cases and 20 controls. The target insecticides that were analyzed in blood were those that corresponded to the insecticides detected in the indoor air samples, and included the parent compounds for *cis*-permethrin and *trans*-permethrin as well as the metabolite of propoxur, 2-isopropoxyphenol.

### Statistical analysis

For both environmental and biologic monitoring data, we assigned samples less than the limit of detection (LOD) a value of 0.5 × LOD. For hypothesis testing, variables were treated as continuous or categorical depending on their distributional properties. Continuous variables were initially log-transformed as appropriate to normalize the distribution. However, in almost all cases, the data could not be normally distributed after log-transformation, so nonparametric statistics were used. The differences in pest infestation levels and air insecticide levels between pre- and postintervention in both cases and controls were normally distributed, so we used parametric statistics to evaluate whether these differences varied significantly between the intervention and control group. Analyses were also undertaken to determine whether the intervention and control groups differed in terms of demographic characteristics or season and year of delivery. No significant differences were seen.

For pest infestation levels, we used the Wilcoxon signed-rank test to assess the differences between pre- and postintervention pest infestation levels in both intervention and control groups. Because the differences in pest infestation levels were normally distributed, we used the independent sample *t*-test to determine whether the differences in pest infestation levels between pre- and postintervention observed in the intervention group were significantly different from the differences in pest infestation levels observed in the control group.

For 2-week integrated air insecticide levels, we compared detection frequencies as well as detection levels. We used McNemar’s test to examine the change in detection frequency of insecticide levels in air between pre- and postintervention in both the intervention and control groups. We used the Wilcoxon signed-rank test to examine the change in insecticide levels between pre- and postintervention for both groups. Finally, we used the independent sample *t*-tests and regression analyses controlling for race/ethnicity, season, and year of delivery to compare whether the change in air insecticide levels between pre- and postintervention differed significantly between the intervention and control groups. For insecticide levels in maternal and cord blood samples, we compared differences in detection frequencies between intervention and control subjects using chi-square analyses (Fisher’s exact test). Results were considered significant at *p* < 0.05 (two-tailed).

## Results

### Study participants

Participants included 25 intervention cases and 27 nonintervention controls. Demographic characteristics were compared between cases and controls and were generally comparable between the two groups (Table 1). Discrepancies between the groups were limited to education and housing conditions (holes in ceilings and walls and leaky pipes) (Pearson’s chi-square test, *p* = 0.008, 0.05, and 0.012, respectively). Table 2 describes reported insecticide use and pest infestation levels in the intervention and control groups. According to the prenatal questionnaire and in compliance with eligibility criteria, 100% of the intervention and control subjects reported exposure to high-toxicity insecticides during pregnancy either through spray by an exterminator, personal (or household) use of spray insecticides, and/or use of a pest bomb. Patterns of insecticide use and reported pest infestation levels were not different between the two groups. Most of both intervention and control subjects reported seeing cockroaches in their homes (91.7 and 85.2%, respectively). More than 50% of both groups reported seeing cockroaches in their homes on a daily basis.

### Pest infestation levels

**Figure 1:**
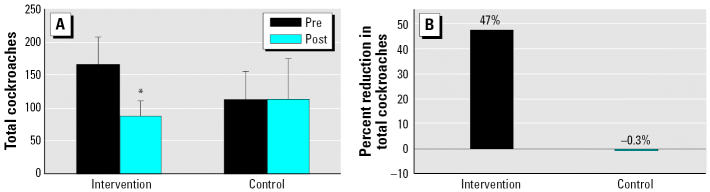
Cockroach infestation levels. (*A*) Mean (± SE) cockroaches (adults + nymphs) in traps collected over 2 weeks pre- (Pre) and postintervention (Post). Intervention group: preintervention (*n* = 22), post-intervention (*n* = 19); control group: preintervention (*n* = 14), postintervention (*n* = 6). (*B*) Percent reduction between total cockroaches in traps collected over 2 weeks pre- and postintervention. Intervention (*n* = 19), control (*n* = 6). Comparison of differences in percent reduction in pest infestation levels between the intervention and control groups, group *t*-test, *p* = 0.1 (*B*). *Wilcoxon signed-rank test, *p* = 0.016.

Pest traps placed in the subjects’ homes for a 2-week collection period before the intervention revealed that adult cockroaches were present at baseline in 77% of the intervention group and 86% of the control group. Figure 1A displays the total number of cockroaches (adult and nymph) collected in traps pre- and postintervention from case and control households. At baseline, the mean (± SE) adult cockroach count was higher in the intervention than in control subjects (27.6 ± 6.8 vs. 11.9 ± 3.1, respectively). At baseline, nymph cockroaches were found in 82% of intervention homes and 93% of control homes and nymph counts were higher in the intervention than in the controls (148.6 ± 32.5 vs. 76.6 ± 21.1). We assessed the effectiveness of the intervention by comparing the differences in pre- and postintervention roach counts within and between intervention and control subjects. Overall, there was a 47% decrease in total cockroach infestation among intervention households after the intervention (Wilcoxon signed-rank test, *p* = 0.016) (Figure 1B). Adult cockroaches decreased by 60% (*p* = 0.006) and nymph cockroaches decreased by 44% (*p* = 0.033). By contrast, control households showed no significant reduction of adult or nymph cockroaches between baseline and follow-up (see Figure 1A). However, the differences between the preintervention compared with postintervention total, nymph, and adult cockroach levels were not significantly greater in the intervention group than in the control group (Figure 1B).

### Air samples

Of the nine insecticides measured in 2-week integrated indoor air samples, five insecticides (bendiocarb, carbaryl, carbo-furan, malathion, and methyl parathion) were not detected or were found in < 10% of either intervention or control samples. Of the remaining insecticides, propoxur was detected in 92% of pre- and postintervention case samples and 100% of baseline and follow-up control samples; and *cis*- and *trans*-permethrin in approximately 30% of pre- and 15% of postintervention samples and 24 and 16% of baseline control samples and 17 and 13% of follow-up control samples, respectively. In addition, piperonyl butoxide was detected in 71% of pre- and postintervention samples and 72 and 57% of baseline and follow-up control samples, respectively. The mean levels for these compounds in pre- and postintervention 2-week integrated indoor air samples are presented in Table 3.

**Figure 2:**
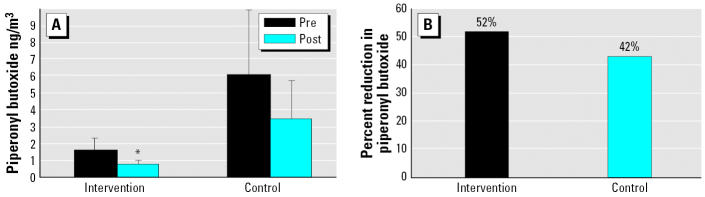
Piperonyl butoxide in 2-week integrated air samples. (*A*) Changes in mean (± SE) piperonyl butoxide levels in 2-week integrated indoor air samples collected over 2 weeks before and 2 weeks after the intervention. Intervention (*n* = 23), control (*n* = 24). (*B*) Percent reduction in piperonyl butoxide levels between 2-week integrated indoor air samples collected over 2 weeks before and 2 weeks after the intervention. Intervention (*n* = 23), control (*n* = 24). Comparison of differences in percent reduction in piperonyl butoxide levels between the intervention and control groups, group *t*-test, *p* = 0.3) (*B*). *Wilcoxon signed-rank test, *p* = 0.016.

Among the intervention group, only piper-onyl butoxide decreased significantly after the intervention (Table 3 and Figure 2A). The mean level of piperonyl butoxide in intervention homes decreased by 50% (Wilcoxon signed-rank test, *p* = 0.016). Of the 23 intervention homes with available air sampling, a decrease in piperonyl butoxide was seen in 74% (17/23) of homes, whereas an increase was seen in 26% (6/23) of homes. Piperonyl butoxide levels also decreased in control homes, but not significantly (*p* = 0.08). The difference between pre- and postintervention levels of piperonyl butoxide in the intervention group was not significantly different from the difference between baseline to follow-up levels in the controls (independent sample *t*-test, *p* = 0.3, Figure 2B). Levels of the pyrethroid insecticides *cis*-permethrin and *trans*-permethrin decreased in follow-up compared with baseline air samples in most intervention homes and increased in most control homes (see negative and positive ranks in Table 3), but these differences were not significant. Propoxur levels decreased nonsignificantly from baseline to follow-up among control homes only (Wilcoxon signed-rank test, *p* = 0.08).

### Biologic samples

Table 4 shows the levels of insecticides that were measured in maternal plasma samples. These included 2-iso-propoxyphenol (metabolite of propoxur) and *cis*- and *trans*-permethrin (two isomers of the pyrethroid insecticide permethrin). These insecticides were detected in plasma samples from the control group but not from the intervention group. Specifically, 2-isopropoxy-phenol was detected in 0% of maternal blood samples from the intervention group and in 12% of maternal blood samples from controls (chi-square, *p =* 0.1). *Cis*- and *trans*-permethrin were detected in 0% of maternal blood samples from intervention group and 12 and 29% of maternal blood samples from controls, differences that were significant for *trans*-permethrin (chi-square, *p =* 0.008) (Table 4). None of these three pesticides were present at levels greater than LOD in either intervention or control cord blood samples.

## Discussion

This pilot intervention study demonstrates that IPM can have a significant effect on pest infestation levels and appears to reduce residential insecticide exposures during pregnancy. Our findings showing significant reductions in cockroach populations are consistent with those of other intervention studies that focused on reducing either pest infestations or allergen levels related to pest infestation (Arbes et al. 2003; Brenner et al. 2003; Kass and Outwater 2001; McConnell et al. 2003; Wood et al. 2001). To our knowledge, however, no other studies have demonstrated reductions in pesticide exposure using biologic and environmental measures of insecticide exposure.

Success of IPM interventions has been attributed to simultaneous application of multiple nonchemical approaches to pest control, including education, repair, least-toxic exterminations, reinforcement, and repetition (Brenner et al. 2003). In our study, attention was focused on problem areas in the house including the kitchen, bathroom, and main living space. In addition to repairing the cracks and holes present in the home, we performed an extensive cleaning to remove food debris, grease stains, and general clutter. Airtight containers were provided for food storage, and individualized education plans were developed for each home that targeted high-risk behaviors. We conclude that the intervention was successful at reducing cockroaches based on data from pest traps placed in the subjects’ homes for 2-week periods immediately before and approximately 1 month after the intervention. Cockroach infestation levels in intervention households declined by more than one-third, whereas cockroach levels in the control households remained unchanged.

Despite the dual goal of IPM to reduce cockroach and insecticide exposures, most IPM evaluations have focused on the reduction of pests. Data on the effectiveness of reducing insecticide exposure are limited, and documented in only two studies. A building-wide intervention in New York City public housing found resident’s use of spray insecticides and Chinese Chalk, an illegal insecticide, dropped to zero after a building-wide IPM intervention that included education about the safe use of insecticides (Kass DE, Outwater T, unpublished data). An IPM intervention in Canada found decreases in both personal use of spray insecticides and resident requests for exterminators to use spray insecticides in their apartment, requesting instead lower-toxicity pastes or gels (Campbell et al. 1999). Although these findings are encouraging, they rely on resident-reported insecticide use after educational sessions and do not include objective measures of insecticide exposures. The current study is the first to use indoor air monitoring and bio-markers to document changes in insecticide exposure after an IPM intervention.

In the present study, target insecticides in indoor air samples included the carbamate, propoxur, the pyrethroids *cis-*permethrin and *trans*-permethrin, and the pyrethroid synergist piperonyl butoxide. Selection of these insecticides was based on evidence that they were widely used for residential pest control (Whyatt et al. 2003). Detection frequencies and mean levels of these insecticides in the current study were similar to those previously documented in this population (Whyatt et al. 2003, 2004). To assess the effectiveness of the IPM, levels of residential insecticides were measured in 2-week integrated indoor air samples collected before and after implementation of the IPM and compared with those for a control population. The effectiveness of the IPM on reducing indoor air levels of residential insecticides can be discerned from trends in piperonyl butoxide. Piperonyl butoxide is a compound added to many pyrethroid formulations to delay metabolic degradation of the active ingredients and enhance insecticidal properties. It is not used in other products and is more volatile than the pyrethroids themselves, so they can be reliably measured in air samples as an indicator of pyrethroid insecticide use. It has been suggested that pyrethroid insecticides are being used to replace the recently restricted organophosphates chlorpyrifos and diazinon (Surgan et al. 2002). In our study, among the intervention households, detection frequencies and levels of piperonyl butoxide decreased significantly after the intervention. This decreasing trend was not significant in the control households. Further, the data suggest that the intervention may have been particularly effective at reducing exposure to the pyrethroid insecticides. Levels of the *trans*-isomer of permethrin were lower in maternal plasma samples collected from the intervention group than in controls.

However, these findings should be interpreted with caution, particularly because results for propoxur do not mirror those seen for piperonyl butoxide. Specifically, propoxur levels in indoor air samples decreased in follow-up compared with baseline air samples among control households, but not among intervention households. Propoxur is a carbamate licensed for residential pest control and has not been subject to regulatory restrictions, as have the organophosphates, chlorpyrifos, and diazinon. However, our prior data suggest that propoxur use in inner-city communities in New York City may be decreasing. Specifically, we found a highly significant decrease in propoxur levels between 1999 and 2001 in personal air samples collected from African-American and Dominican women in New York City during pregnancy and in the corresponding blood samples collected from the mothers and newborns at delivery (Whyatt et al. 2003, 2004). Unfortunately, no data are available comparing frequencies of pyrethroid versus propoxur use in these communities.

Although this pilot intervention indicated that IPM is effective at reducing pest infestation and the internal dose of the insecticides during pregnancy, limitations in the study design should be noted. The primary limitation of the study is the small sample size and the short time elapsed between pre- and postintervention monitoring. Many intervention studies allow 6 months to 1 year between samplings to determine if the intervention is both successful and sustainable. However, the current intervention was conducted during pregnancy and was thus limited in follow-up time. Further, the controls were selected from an ongoing biomarker validation study that followed women only during pregnancy. Thus we were not able to evaluate the sustainability of the intervention over an extended period. In addition, the optimal design for an intervention study is to match the intervention subjects to control subjects and have all data collected and analyzed simultaneously. This was not possible here because the controls were selected from ongoing research. However, the intervention and control groups were comparable in terms of years of enrollment and self-reported pesticide use.

A principal goal of the pilot study was to assess whether environmental and biologic measures can be used in evaluating the efficacy of IPM interventions in reducing residential pesticide exposures. These initial results are promising, although additional research is warranted given the small sample size and inconsistency in some of the findings. Environmental measures for the targeted pesticides are not necessarily associated with the biologic measures. Therefore, a lack of meaningfully different results in air levels of pesticides between the intervention and control groups does not influence the expected results in maternal plasma between the two groups. Subsequent research could also draw on our study design to devise an IPM intervention that can be conducted by household members themselves and that is both feasible and affordable. Such an intervention could be applied to entire apartment buildings or complexes to determine the effects of larger-scale interventions, as opposed to individual units. In the current study, cleaning and home repairs were completed by a professional cleaning crew to allow comparability and consistency. However, the supplies and techniques are similar to those available in the community. In conclusion, we believe that this intervention protocol using IPM can be successfully adapted for use by individuals within households in this community to reduce pest infestation levels and residential pesticide exposure.

## Figures and Tables

**Table 1:** Distribution of maternal sociodemographic characteristics gathered from questionnaires administered on recruitment (intervention group, *n* = 25; control group, *n* = 27).

Characteristic	Intervention (%)	Control (%)
Age [years (range)]	26.6 (19–36)	24.3 (18–36)
Education^a^		
< High school diploma	28.0	55.6
High school diploma or equivalent	28.0	37
> High school diploma	44.0	7.4
Race/ethnicity		
Latina	76.0	59.3
African American	20.0	37.0
Marital status		
Married	12.0	22.0
Never married	72.0	66.7
Divorced/separated	16.0	11.1
Income		
< $10,000	44.0	41.7
$10,000–30,000	40.0	45.8
> $30,000	12.0	12.3
Housing conditions		
Holes in ceiling/walls^a^	75.0	48.1
Unrepaired water damage	40.0	22.2
Leaky pipes^a^	42.0	11.1
Year of delivery		
2002	28.0	44.4
2003	68.0	33.3
2004	4.0	22.2
Season of delivery		
January–March	20	30.8
April–June	8	23.1
July–September	40	15.4
October–December	32	30.8

Missing information for the two groups includes, for intervention, race/ethnicity (1), income (1), unrepaired water damage (1), leaky pipes (1); for control, income (3), season of delivery (1).

a Pearson’s chi-square test, *p* < 0.05.

**Table 2:** Reported pest infestation levels and pesticide use assessed from prenatal questionnaires administered on recruitment (intervention group, *n* = 25; control group, *n* = 27).

	Affirmative (%)	
Questionnaire	Intervention	Control
Reported roaches	91.7	85.2
Reported exposure to high-toxicity pest control measures:	100	100
Spray by exterminator	47.8	40.7
Can spray	73.9	76.9
Pest bomb	31.8	15.4

Missing information for the two groups includes, for intervention, reported roaches (1), spray by exterminator (2), can spray (2), pest bomb (2); for control, can spray (1), pest bomb (1).

**Table 3:** Pre- and postintervention levels of pesticides measured in 2-week integrated indoor air samples (ng/m^3^) collected from intervention group and control group from African-American and Latina subjects residing in northern Manhattan and the South Bronx.

		Intervention (*n* = 25)						Control (*n* = 24)					
				Wilcoxon signed-rank test						Wilcoxon signed-rank test			
Pesticide	LOD (ng/m^3^)	Preintervention (mean ± SE)	Postintervention (mean ± SE)	Negative ranks^a^	Positive ranks^b^	Ties	*p*-Value	Preintervention (mean ± SE)	Postintervention (mean ± SE)	Negative ranks^a^	Positive ranks^b^	Ties	*p*-Value
Propoxur	0.2	49.3 ± 19.0	56.8 ± 24.2	11	11	0	0.592	45.6 ± 10.9	36.9 ± 8.3	14	10	0	0.081
Piperonyl butoxide	0.2	1.66 ± 0.71	0.80 ± 0.22	17	6	0	0.016	6.12 ± 3.8	3.5 ± 2.2	14	9	1	0.089
*cis-*Permethrin	0.4	1.54 ± 0.85	1.25 ± 0.60	14	11	1	0.510	1.84 ± 1.48	0.99 ± 0.63	9	13	2	0.758
*trans*-Permethrin	0.7	2.60 ± 1.45	1.9 ± 0.96	13	11	0	0.475	2.75 ± 2.2	1.66 ± 1.0	9	15	0	0.338

Missing information includes, for intervention, propoxur (3), piperonyl butoxide (2), *trans*-permethrin (1).

a Levels were lower in follow-up compared with baseline.

b Levels were higher in follow-up compared with baseline.

**Table 4:** Pesticide levels (pg/g) in maternal plasma samples collected at delivery in intervention group (*n* = 21) and in control group (*n* = 17).

Pesticide	LOD	Intervention (% > LOD)	Control (% > LOD)	Chi-square *p*-value^a^
2-Isopropoxyphenol	1.50	0	11.8	0.106
*cis-*Permethrin	0.50	0	11.8	0.106
*trans*-Permethrin	0.50	0	29.4	0.008

Missing information includes, for controls, *cis*-permethrin (1), *trans*-permethrin (1).

a Difference in detection frequency between intervention and control groups.
